# Medical Department Pan-American Exposition

**Published:** 1902-01

**Authors:** 


### Medical Department Pan-American Exposition.

A YEAR ago—January, 1901—we presented to our readers a very complete illustrated description of the great fair to open in Buffalo, May 1, 1901, and to continue for six months. From time to time during the year just ended we have published such material, reports, and items as we deemed of special interest to readers of the JOURNAL. Elsewhere in this issue will be found the comprehensive report of Dr. Roswell Park, Medical Director of the Exposition, which is a befitting finale to the literature pertaining to the fair,—the last we expect to print.

We take pleasure in publishing this report because (1) it will be found interesting reading as are all of Dr. Park's writings; (2) it is a most creditable history of a great medical supervision conducted at a minimum of cost in life and money; (3) its unsolicited and unrestricted use was offered primarily to the JOURNAL (too often such reports first appear in the newspapers); and (4) it is the one bright spot in the conduct of the exposition,—a department in which there was no scandal, but only unremitting attention to duty, with scientific application, and with splendid results.

In reading this report one cannot but be impressed with its simplicity and completeness, and also with the wisdom of the Medical Director in establishing, even against great opposition, an admirable hospital, completely equipped, and that the entire medical control of hospital and sanitary affairs of the exposition was merged in a single executive.

